# The Effect of Transesophageal Echocardiogram on Clinical Outcomes for Patients with *Staphylococcus aureus* Bloodstream Infection

**DOI:** 10.3390/antibiotics15020159

**Published:** 2026-02-03

**Authors:** Hiba Al Shaikhli, Mary Joyce B. Wingler, Kayla R. Stover, Katie E. Barber, Jamie L. Wagner, David A. Cretella

**Affiliations:** 1 Department of Antimicrobial Stewardship, University of Mississippi Medical Center, Jackson, MS 39216, USA; hialshai@utmb.edu (H.A.S.); mwingler@umc.edu (M.J.B.W.); dcretella@umc.edu (D.A.C.); 2 Department of Pharmacy Practice, University of Mississippi School of Pharmacy, Jackson, MS 39216, USA; kbarber@umc.edu (K.E.B.);

**Keywords:** Gram-positive infection, infective endocarditis, predictive scores, *Staphylococcus aureus* bacteremia

## Abstract

**Background/Objectives**: *Staphylococcus aureus* bloodstream infections (SABSIs) are associated with significant morbidity and mortality and are often complicated by infective endocarditis (IE). During the COVID-19 pandemic, fewer transesophageal echocardiographs (TEEs) were performed for patients diagnosed with SABSI, and this study examined the impact on clinical outcomes associated with this change in practice. **Methods**: This retrospective observational study included adult patients treated for SABSI who were admitted pre-COVID-19 (1 March 2018 to 11 March 2020) and during COVID-19 (12 March 2020 to 1 March 2022). Primary outcomes were rates of confirmed IE and duration of antibiotic therapy (DOT). **Results**: Of 333 screened patients, 214 were included (107 per group). Patients in the COVID-19 group were older (51.49 vs. 56.31 years, *p* = 0.013); other baseline characteristics were similar. Catheter-related infections were the most common source in the pre-COVID-19 and COVID-19 groups (30.8% vs. 18.9%, *p* = 0.089). Rates of TEE procedures significantly declined during COVID-19 (72% vs. 50.9%, *p* = 0.002); rate of confirmed IE (9.4% vs. 12.1%; *p* = 0.660) and median DOT (28 vs. 28 days; *p* = 0.596) were similar. Ninety-day mortality was higher in the COVID-19 group (10.4% vs. 22.2%, *p* = 0.019); other outcomes were not statistically different. **Conclusions**: The COVID-19 pandemic led to a notable decline in TEEs performed for SABSI, but the majority of clinical outcomes were unchanged. Mortality was significantly higher in the COVID-19 group, but it is uncertain that this was solely due to the change in practices. In a healthcare system that universally recommends TEE, scoring systems may help identify which patients are highest priority for TEE versus those that could undergo a transthoracic echocardiogram.

## 1. Introduction

*Staphylococcus aureus* is a gram-positive bacterium that causes many community- and healthcare-associated infections, including bloodstream infections (SABSI). SABSIs are associated with up to 30% mortality, and complications are common [1,2,3,4]. Infective endocarditis (IE) is one of the most serious complications and occurs in 6.5 to 20% of patients with SABSI [1,5]. Due to the high incidence of IE, echocardiography should be performed to visualize any potential intracardiac masses, vegetations, abscesses, valve dehiscence, and valvular regurgitation [1]. The Infectious Diseases Society of America (IDSA) guidelines for the treatment of methicillin-resistant *Staphylococcus aureus* (MRSA) infections recommend performing echocardiography for all adult patients presenting with a bloodstream infection [6]. A transesophageal echocardiography (TEE) is preferred over transthoracic echocardiography (TTE) due to superiority of the former for identifying IE [6,7]. The American Heart Association (AHA) guidelines recommend TTE followed by TEE in all patients at high risk of IE, such as prosthetic heart valves, certain congenital heart diseases, a history of endocarditis, or a new heart murmur or heart failure [8]. Despite these recommendations, only 19% of physicians perform a TEE following a negative TTE result, according to a journal article assessing clinical practice variation among infectious disease physicians in the management of *Staphylococcus aureus* bacteremia (SAB). However, the majority, 93%, of providers performed a TTE initially [9].

Despite the diagnostic advantages, TEE is associated with higher costs and potential risks, and many patients are not suitable candidates for immediate or subsequent TEE due to various factors, including clinical stability or physical injuries to the esophageal space [10]. The AHA guidelines suggest that a TTE alone may be sufficient for patients who are at low risk with low clinical suspicion for IE; however, identifying this low-risk group remains challenging [8]. Previous studies have described certain possible indicators of a low risk of IE, including the nosocomial acquisition of bacteremia, sterile follow-up blood cultures within 96 h, the absence of permanent cardiac devices, no dependence on hemodialysis, and a lack of clinical signs of endocarditis or secondary foci of infection [1]. Several scores have been developed to aid in distinguishing patients who are at high or low risk for IE due to SABSI, including the POSITIVE (the Prediction Of Staphylococcus aureus Infective endocarditis Time to positivity, IV drug use, Vascular phenomena, preExisting heart condition), PREDICT (Predicting Risk of Endocarditis Using a Clinical Tool), and VIRSTA scores (Table 1) [5,11,12,13,14]. These tools have the potential to more easily stratify patients who should undergo a TEE procedure, preserving healthcare resources and decreasing risk of procedural-related complications, such as esophageal rupture, which occurs in about 1 in every 5000 cases [3].

In patients with COVID-19, an estimated 3–10 cases of infective endocarditis per 100,000 population have been reported [15]. Prior to the COVID-19 pandemic, the majority of patients with SABSI at this institution underwent TEE to rule out endocarditis. However, during the pandemic, fewer patients with SABSI underwent TEE procedures due to unstable clinical conditions and concerns for infection transmission. In addition, there was no change to other advanced cardiac imaging techniques in place of TEE, such as ^18^F-fluorodeoxyglucose (FDG)-positron emission tomography (PET)/computed tomography (CT). Therefore, this study aims to explore changes in clinical decision making and associated clinical outcomes for patients with SABSI before and during the COVID-19 pandemic.

## 2. Results

### 2.1. Baseline Demographics

**Figure 1:**
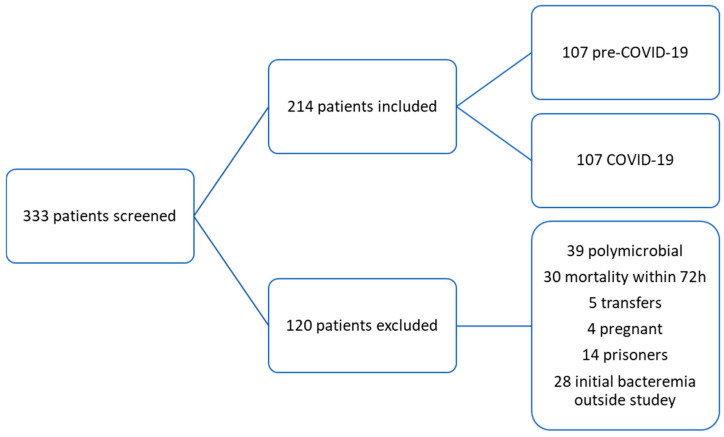
Enrollment.

A total of 333 patients were screened, and 214 patients were included in this study with 107 allocated to each group (Figure 1). The most common reasons for exclusion included polymicrobial BSI (*n* = 39), mortality within 72 h of BSI (*n* = 30), and initial BSI outside of the study date parameters (*n* = 28).

**Figure 2:**
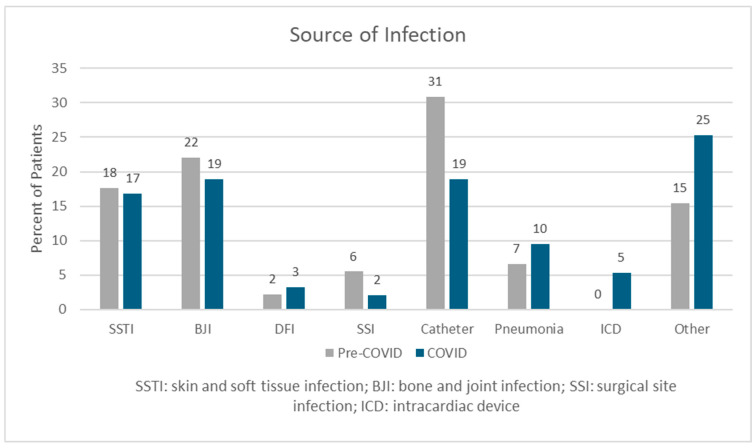
Sources of infection.

Patients in the COVID-19 group were significantly older than those in the pre-COVID-19 group (51.49 vs. 56.31 years old; *p* = 0.013); other baseline characteristics were similar between groups (Table 2). Most patients in both groups presented with a community-acquired infection (80.2% vs. 80.6%; *p* = 0.946), and there were no differences in presence of risk factors for IE, such as history of intravenous drug use (IVDU), cardiac valve replacement, or intracardiac devices. More patients in the COVID-19 group required mechanical ventilation (12.6% vs. 18.5%; *p* = 0.238) and vasopressors (8.7% vs. 16.7%, *p* = 0.85). Of the 93 patients tested for COVID-19, only 11% had a confirmed positive test. The most common source of infection was catheter-related infections (31% vs. 19%; *p* = 0.089) (Figure 2). Most patients classified as having “other” infection sources had multiple suspected or confirmed foci.

### 2.2. SABSI Management

Most patients in both groups were followed by the infectious diseases (ID) consultation service (95.4% vs. 99.1%; *p* = 0.213). The majority of patients had a TTE performed in the pre-COVID and COVID-19 groups (93.4% vs. 92.5%; *p* = 0.789). However, there was a notable decline in the number of TEE procedures in the COVID-19 group compared to the pre-COVID-19 group (50.9% vs. 72%; *p*-value = 0.002) (Table 3).

### 2.3. Outcomes

Rate of confirmed IE was not significantly different between the pre-COVID-19 and COVID-19 groups (9.4% vs. 12.1%; *p* = 0.658). The median duration of antibiotic therapy was 28 days (interquartile range [IQR] 28–42) in both groups (*p* = 0.588). The 90-day mortality rate was higher in the COVID-19 group compared to the pre-COVID-19 group (22.2% vs. 10.4%; *p* = 0.019). In the COVID-19 group, mortality was not significantly higher in patients who tested positive for COVID-19 compared with those who tested negative (4/12 [33%] vs. 20/81 [25%]). There was no difference between other secondary outcomes (Table 3 and Table 4).

The majority of patients had a high VIRSTA score in both groups (Table 4). However, fewer patients with a high VIRSTA score (≥3) had a TEE completed in the COVID-19 group (94% vs. 72%; *p* = 0.076).

## 3. Discussion

Our study identified that the change in echocardiography practices in patients with SABSI during the COVID-19 pandemic did not significantly change most clinical outcomes, including rate of confirmed IE and antibiotic durations. Mortality was higher in the COVID-19 group, but this may not have been due to the decline in TEE alone. The results of this study highlight opportunities to implement risk stratification to identify patients who may not require a TEE, which could lead to cost savings for patients and the healthcare system.

It is well established that TEE has better sensitivity than TTE for diagnosing IE, and rates of TEEs in previous studies typically fall between 30% and 50% [6,16,17]. At this institution, a SABSI intervention was implemented in 2014 by Brock and colleagues, which encouraged changes to antibiotic-prescribing practices, source control procedures, and universal TEE [18]. Focusing on echocardiography, the intervention resulted in an increased rate of TEEs performed from 25.5% to 54.8% in the pre- and post-groups, respectively. The pre-group of the current study included patients admitted approximately two to four years after the previous publication, and TEE rates had continued to rise to 72%. Though fewer TEE procedures were performed during the pandemic, the rate remains above that of many healthcare systems and did not appear to negatively impact clinical outcomes. In addition, IE rates from the previous study were 8.1% and 12% before and after the SABSI intervention, respectively, which fall within the normal range reported in the literature [18]. This is similar to the results of the present study with 9.4% in the pre-COVID-19 group and 12.1% in the COVID-19 group being diagnosed with IE. However, it is important to note that there are factors that can impact the diagnostic performance of echocardiography. For example, TEE may be preferred in patients with central obesity due to poor image quality or in patients with ICD or prosthetic valves due to low sensitivity and specificity [19,20]. Although we did not specifically assess obesity, the range of weights of our patient population suggests that some may meet the definition and be qualified as obese. This may have impacted positive results in this population. The current study did not have a large population of the latter group of patients, so we expect the impact on results to be minimal.

However, not all patients with SABSI can undergo the TEE procedure. Changes in echocardiography rates due to the COVID-19 pandemic allowed for reflection on how to identify patients who were at highest need of a TEE to rule out IE. During the study period, none of the three scoring systems, POSITIVE, PREDICT, or VIRSTA, were used for risk stratification; however, this study demonstrated the impact that this potentially could have on utilization of healthcare resources. All three scoring systems are user-friendly with a limited number of items to answer, and all data points should be readily available in a patient’s medical record [5,11,12]. This study used the VIRSTA score based on results from a comparison of the three options performed by van der Vaart and colleagues [16]. Results demonstrated that the VIRSTA score has the highest negative predictive value (99.3%) compared with the POSITIVE (92.5%) and PREDICT (94.5%). This may be because the VIRSTA score accounts for more patient-specific factors than the other prediction scores, including the presence of cerebral/peripheral emboli, meningitis, intracardiac devices, previous IE, preexisting native valve disease, persistent bacteremia, intravenous drug use (IVDU), vertebral osteomyelitis, severe sepsis or shock, and C-reactive protein levels. A VIRSTA score can range from zero to 28, and a result of three or more indicates a patient at high risk of IE, and a TEE should be performed. As the VIRSTA score increases so does the risk. In this study, most patients with a high VIRSTA score had a TEE completed in both groups though there was a decline in the COVID-19 group (94% vs. 72%). Additionally, 22/23 (95.6%) of patients with confirmed IE had a VIRSTA score of 5 or more. On the other hand, 35% and 31% of patients in the pre-COVID-19 and COVID-19 groups had a low VIRSTA score, and most (65.7% vs. 58.8%) had a TEE performed. These findings demonstrate implementation of the VIRSTA score could provide additional support for TEE in high-risk patients and identify patients in whom a TTE alone would be adequate.

Duration of antibiotic therapy was originally hypothesized to have increased to six weeks during the COVID-19 pandemic due to the decline in TEE and inability to rule out IE; however, results of this study demonstrated patients received approximately a month of antibiotics in both groups. According to the 2011 IDSA MRSA guidelines, the duration of antibiotic therapy for SABSI is dependent on if the patient meets criteria for uncomplicated versus complicated infection [6,17]. Though the definitions continue to be debated amongst ID providers, uncomplicated SABSI has conventionally been defined as exclusion of endocarditis and other metastatic sites of infection, lack of prostheses, negative blood cultures by 96 h, and defervescence within 72 h of antibiotic initiation; the recommended duration of antibiotic therapy is 14 days for patients who meet these criteria [6,9,21]. For those who are considered complicated, a duration of four to six weeks is recommended. Though the current evaluation did not assess the percentage of patients classified as complicated versus uncomplicated, the previous study by Brock and colleagues found 5% of patients at this institution met criteria for an uncomplicated SABSI [18]. In this study, 10 patients (9.3%) in the pre-COVID-19 group and 12 (11.2%) in the COVID-19 group received 14 days of therapy or less, suggesting that the rate of uncomplicated SABSI remains low. This rate of uncomplicated infection is similar to other publications. Taupin and colleagues conducted a study evaluating uncomplicated SABSI durations, and of the 612 patients with a positive culture for *S. aureus* during the study period, only 64 (10%) could be included [22]. The majority of patients who were excluded were excluded due to factors that would classify the patient as a complicated SABSI. Randomized controlled trials for uncomplicated SABSI have historically suffered from low enrollment rates. For example, the SABATO trial screened 5063 patients and only 213 (4.2%) were included [23]. The authors discussed a typical screening-to-enrollment ratio for uncomplicated SABSI was 1:25, and emphasized the challenges associated with categorizing patients as “low risk”. Because most patients had a complicated infection in the current study, the median of 28 days is appropriate based on current definitions and guidelines. Considering the rates of IE and complicated SABSI, it is reasonable to re-evaluate universal TEE at this institution.

Mortality associated with SABSI remains high, but rates may have improved in the last 20 years [24]. Bai and colleagues performed a meta-analysis evaluating mortality due to SABSI in three time periods: prior to 2001, 2001–2010, and 2011 onward [24]. For both MRSA and MSSA bloodstream infections, 30-day mortality was lower in the latter time periods (MRSA: 33.4% vs. 25.9% vs. 23.4%; *p* = 0.0016; MSSA: 22.5% vs. 17.1% vs. 18.6%; *p* = 0.0739), but 90-day mortality was not significantly changed. In the current study, 90-day mortality was significantly lower in the pre-COVID-19 group compared with the COVID-19 group (10.4% vs. 22.2%; *p* = 0.019) and also when compared to the most previous literature. Thirty-day mortality was not assessed as we expected most patients to still be receiving therapy. There was no significant difference between patients who tested positive for COVID-19 and those who did not, indicating it may not have been due to the infection alone. However, the pandemic had a negative impact on healthcare resources and patient care, which could have led to the increase seen in the COVID-19 group. Based on the data available, it is unclear what led to the increase in mortality in this study, and it should be explored again with the resumption of normal day-to-day operations.

This study is not without limitations. As a retrospective, single-center study, generalizability to other healthcare systems is decreased. As described, the classification of uncomplicated versus complicated SABSI was not collected, nor was a disease severity score (ex. Pitt bacteremia score). This limits the ability to fully assess the mortality difference between groups. Furthermore, the rationale for duration was not collected, and echocardiography alone was likely not the only driver in clinical decision making. In addition, follow-up blood culture timing was inconsistent, which could have impacted the duration of positive blood cultures and, in turn, the antibiotic duration of therapy. There was also a limited number of patients with COVID-19, which may have lessened the impact of the pandemic upon clinical outcomes in the COVID-19 group. The positive COVID-19 sample size may also be a confounder impacting the 90-day mortality. Next, changes to antibiotics after discharge could not be accurately captured; therefore, duration was based on what was planned during the hospitalization. Finally, although there was no difference in the IE incidence rate and VIRSTA score, this retrospective trial does not evaluate all outcomes associated with TEE. Further verification is needed to generalize these findings.

## 4. Materials and Methods

### 4.1. Study Design

This retrospective, observational, pre/post study evaluated adult patients treated for SABSI at an academic medical center. Patients were divided into two groups: pre-COVID-19 (1 March 2018 to 11 March 2020) and COVID-19 (12 March 2020 to 1 March 2022). Dates were selected based on two years before and after the date of the first COVID-19 patient admitted to this institution. Patients were excluded if they met any of the following criteria: polymicrobial infection, mortality within 72 h of the first positive blood culture, transferred from external medical facilities after receiving over 72 h of antibiotic therapy for SABSI, or pregnant or incarcerated during the time of evaluation. If patients had multiple episodes of SABSI during the study period, only the initial incidence was included. Data collection included basic patient demographics, microbiological data, a calculated VIRSTA score, and antimicrobial utilization data. Data was collected and stored in REDCap (Research Electronic Data Capture) [25].

### 4.2. Outcomes

Primary outcomes were the rate of confirmed IE and the duration of antibiotic therapy prescribed in the pre-COVID-19 and COVID-19 groups. Secondary outcomes included rates of treatment failure, hospital length of stay, outpatient antibiotic services (OAS) consultation rate, correlation between patient VIRSTA score and TEE procedure, rate of 90-day mortality, and rate of 60-day re-admission. The VIRSTA score was chosen over POSITIVE and PREDICT due to having the highest negative predictive value among the three risk stratification tools [16].

### 4.3. Definitions

Community-acquired infections were those in which blood cultures were positive within 48 h of admission. Infective endocarditis was defined as presence of a vegetation confirmed on echocardiography plus positive cultures. Antimicrobial days were calculated as the aggregate sum of days for which any amount of a specific antimicrobial agent was administered that covered the pathogen, including planned outpatient days of therapy. Escalation of antimicrobial therapy was defined as antibiotics changed from monotherapy to combination therapy (ex. daptomycin and ceftaroline for methicillin-resistant *S. aureus* [MRSA] or cefazolin and ertapenem for methicillin-susceptible *S. aureus* [MSSA]). Treatment failure was defined as a composite of all-cause 90-day mortality, escalation in antimicrobial therapy, or all-cause re-admission within 60 days.

### 4.4. Statistical Analysis

Data were analyzed using SPSS (version 28.0, IBM, Armonk, NY, USA). A target sample size of at least 214 patients was determined to provide 80% statistical power for detecting a 20% increase in therapy duration and a 35% increase in mortality. Therapy duration was reported at 35 ± 14 days with an estimated increase of 20% [1,4], and mortality was reported at 54% with an estimated absolute decrease of 35% [1,5]. Categorical variables were assessed using the chi-squared test or Fisher’s exact test, while continuous variables were analyzed using the Mann–Whitney U test or Student’s *t*-test. SPSS was used to assess the normal distribution of data. A *p*-value less than 0.05 was considered indicative of statistical significance.

## 5. Conclusions

This study evaluated how the COVID-19 pandemic changed echocardiography practices in patients with SABSI. The findings confirmed a significant decline in TEE procedures performed in SABSI patients in the COVID-19 group but found no change in rates of IE diagnoses or durations of antibiotic therapy. Ninety-day mortality was higher in the COVID-19 group, but it is unclear what caused this increase. Integrating tools like the VIRSTA score into routine clinical practice may improve the identification of patients who could have a TTE alone, thereby potentially preserving healthcare resources and decreasing risk of complications due to TEE procedures.

## Figures and Tables

**Table 1:** Components of the three major endocarditis prediction scores.

	VIRSTA	Points Allotted	POSITIVE	Points Allotted	PREDICT	Points Allotted
Culture-related factors			TTP < 9 h	5	Positive culture after 72 h	2
			TTP 9–11 h	3		
			TTP 11–13 h	2		
Laboratory markers	C-reactive protein > 190 mg/L	1				
Acquisition	Community or non-nosocomial healthcare-associated acquisition	2			Community acquisition	2
					Healthcare acquisition	1
Presentation	Cerebral or peripheral emboli	5	Vascular phenomena	6		
	Meningitis	5				
	Persistent bacteremia	4				
	Vertebral osteomyelitis	2				
	Severe sepsis or shock	1				
Comorbidities	Permanent intracardiac device or previous IE	4	IV drug use	3	ICD	2
	Pre-existing native valve disease	3	Predisposing heart disease	5	Permanent pacemaker	3
	Intravenous drug use	4				
Score Interpretation		Cutoff: ≥3 = high risk for IE		Cutoff: >4 = high risk for IE		Cutoff: ≥2 (day 5 score) = high risk for IE

ICD—intracardiac device; IE—infective endocarditis; IV—intravenous; TTP—time to positivity.

**Table 2:** Baseline characteristics.

Variable, Median (IQR) or *n* (%)	Pre-COVID-19 (*n* = 107)	COVID-19 (*n* = 107)	*p*-Value
Age (years), mean (SD)	51.49 (13.77)	56.31 (13.96)	0.013
Weight (kg)	80.7 (67.5–100.7)	81.6 (65.3–106.0)	0.600
Gender (male)	73 (68.9)	71 (65.7)	0.626
Black	55 (51.9)	54 (50.0)	0.786
Charlson Comorbidity Index	4 (2–6)	5 (2–6)	0.081
Central line access at the time of SABSI identification	34 (31.1)	35 (32.4)	0.959
History of IVDU	13 (12.3)	8 (7.4)	0.230
Current	7 (53.8)	5 (62.5)	1.000
History of IE	1 (0.9)	0 (0)	0.4953
Valve replacement	3 (2.9)	2 (1.9)	1.000
Native valve disease	12 (11.3)	7 (6.5)	0.238
Intracardiac device	6 (5.7)	10 (9.3)	0.317
Hemodialysis	20 (18.9)	24 (22.2)	0.544
COVID-19	--	*n* = 93	0.004
		12 (11)	
Febrile	63 (60)	56 (51.9)	0.231
Mechanical Ventilation	13 (12.6)	20 (18.5)	0.238
Vasopressors	9 (8.7)	18 (16.7)	0.85
Community-acquired	85 (80.2)	87 (80.6)	0.946
MSSA	47 (44.3)	52 (48.1)	0.576

IVDU = Intravenous drug use; IE = Infective endocarditis; MSSA = Methicillin-susceptible Staphylococcus aureus.

**Table 3:** Management.

Variable, Median (IQR) or *n* (%)	Pre-COVID-19 (*n* = 107)	COVID-19 (*n* = 107)	*p*-Value
Source control *	64 (60.4)	57 (52.8)	0.047
ID consult	103 (99.1)	103 (95.4)	0.213
LOS	13 (11–19)	11 (8–20)	0.910
Definitive antibiotics			
Cefazolin	39 (36.8)	43 (39.8)	0.649
Nafcillin	8 (7.50)	6 (5.60)	0.556
Vancomycin	50 (47.2)	43 (39.8)	0.278
Daptomycin	7 (6.60)	10 (9.30)	0.473
Other	2 (1.90)	7 (6.50)	0.094
TTE	99 (93.4)	98 (92.5)	0.789
TEE	76 (72)	55 (50.9)	0.002

ID = infectious diseases; LOS = length of stay; TTE = transthoracic echocardiogram; TEE = transesophageal echocardiogram; * source control measures included: catheter removal, incision and debridement/drainage, bone resection/amputation or device removal.

**Table 4:** Clinical outcomes.

Variable, Median (IQR) or *n* (%)	Pre-COVID-19 (*n* = 107)	COVID-19 (*n* = 107)	*p*-Value
Confirmed IE	10 (9.4)	13 (12.1)	0.660
Antibiotic duration in days	28 (28–42)	28 (28–42)	0.596
OAS consults	38 (35.8)	36 (33.3)	0.774
Length of stay	13 (11–19)	11 (8–20)	0.91
90-day mortality	11 (10.4)	24 (22.2)	0.019
60-day readmission	5 (4.80)	4 (3.70)	0.701
Antibiotic escalation	8 (7.60)	10 (9.50)	0.622
Treatment failure *	20 (18.9)	31 (28.7)	0.91
High VIRSTA score (≥3)	69 (64.5)	73 (68.2)	0.664
TEE performed	65/69 (94.2)	53/73 (72.6)	0.076
TEE not performed	4/69 (5.8)	20 (27.4)	0.0008
Low VIRSTA score (<3)	38 (35.5)	34 (31.8)	0.664
TEE performed	25/38 (65.8)	20/34 (58.8)	0.404
TEE not performed	13/38 (34.2)	14/34 (41.2)	0.682

* Composite of 90-day mortality, 60-day readmission, and antibiotic escalation; IE = infective endocarditis; OAS = outpatient antibiotic service; TEE = transesophageal echocardiogram.

## Data Availability

The original contributions presented in this study are included in the article. Further inquiries can be directed to the corresponding author.

## Abbreviations

The following abbreviations are used in this manuscript:

AHA	American Heart Association
CT	Computed tomography
IDSA	Infectious Diseases Society of America
IE	Infective endocarditis
IVDU	Intravenous drug use
MRSA	Methicillin-resistant *Staphylococcus aureus*
MSSA	Methicillin-susceptible *Staphylococcus aureus*
PET	Positron emission tomography
POSITIVE	Prediction Of *Staphylococcus aureus* Infective endocarditis Time to positivity, IV drug use, Vascular phenomena, preExisting heart condition
PREDICT	Predicting Risk of Endocarditis Using a Clinical Tool
SAB	*Staphylococcus aureus* bacteremia
SABSI	*Staphylococcus aureus* bloodstream infection
TEE	Transesophageal echocardiograph
TTE	Transthoracic echocardiograph
